# 

**Published:** 1882-07

**Authors:** 


					﻿ESTABLISHED IN 1BF5K
vS"xx-no.<.	JULY, 1882.	v’lTiX'ft
ATLANTA
MEDICAL REGISTER.
IA TLAN TA MEDIC A L A NI) S Uli GICA L JO URN A L.)
EDITED ~~Z7
JAMES B. BAIRD, M.D.
H. H. DICKSON, PUBLISHER. TERMS, $2.50 A YEAR IN ADVANCE
ATLANTA, GEORGIA':
Z/. II. DICK SOK, BOOK AKD JOB PBIKTBB AKD BOOKBIKDFB,
1882.
				

